# PD23 Analysis Of The Critical Assessment Reports Of Technologies Incorporated By The National Supplementary Health Agency

**DOI:** 10.1017/S026646232510158X

**Published:** 2025-12-29

**Authors:** Vanelise Zortea, Isabelle de Lima dos Santos, Letícia Brust, Maria Angélica Pires Ferreira

## Abstract

**Introduction:**

As a step in the process of incorporating technologies, the National Supplementary Health Agency (ANS) prepares critical assessment reports (RACs). The analysis of the RACs can help in understanding the demands in supplementary health. The aim of this work was to characterize the technologies submitted to and incorporated into the ANS with respect to type of intervention, certainty of evidence, and economic aspects.

**Methods:**

A descriptive study was conducted in which data from the RACs of the proposals for updating the list of covered procedures, incorporated between October 2021 and December 2023, were analyzed. The extracted data included type of intervention; classification of the disease or condition; level of certainty of the evidence for the main outcome; incremental cost-effectiveness ratio (ICER); and incremental budget impact (IBI) over five years. For the ICER evaluation, the percentage of technologies where the cost exceeded three times the Brazilian gross domestic product (GDP) for 2023 (USD31,103.4557) was considered. Data were presented using descriptive statistics.

**Results:**

Of the 50 incorporated technologies, 96 percent were therapeutic and 4 percent were preventive or prophylactic. Most of the proposals were for oncological conditions (68%). The certainty of the evidence was moderate to high in 52 percent of the incorporations and low to very low in 48 percent. The average IBI was USD32,001,783.74. In 24 percent of the incorporations, the IBI was negative, with 75 percent of these having low to very low evidence certainty. A cost-minimization analysis was conducted for 15 proposals; for the others, the ICER was more than three times the GDP in 54.3 percent of the incorporations.

**Conclusions:**

A relevant percentage of the technologies incorporated by the ANS had low to very low certainty of evidence. Costs exceeded three times the Brazilian GDP in more than half the technologies where the ICER was calculated. The results highlight the need for decisions based on robust evidence and cost effectiveness to ensure the sustainability of the supplementary health system.

